# Regulation of *TSHR* Expression in the Thyroid and Thymus May Contribute to TSHR Tolerance Failure in Graves' Disease Patients via Two Distinct Mechanisms

**DOI:** 10.3389/fimmu.2019.01695

**Published:** 2019-07-18

**Authors:** Ana Marín-Sánchez, Daniel Álvarez-Sierra, Oscar González, Ana Lucas-Martin, Alicia Sellés-Sánchez, Francesc Rudilla, Emma Enrich, Roger Colobran, Ricardo Pujol-Borrell

**Affiliations:** ^1^Immunology Division, FOCIS Center of Excellence, Hospital Universitari Vall d'Hebron, Barcelona, Spain; ^2^Diagnostic Immunology Group, Vall d'Hebron Research Institute, Barcelona, Spain; ^3^Department of Cell Biology, Physiology and Immunology, Universitat Autònoma de Barcelona, Barcelona, Spain; ^4^Surgery Department, Hospital Universitari Vall d'Hebron, Barcelona, Spain; ^5^Endocrinology Division, Hospital Universitari Germans Trias Pujol, Badalona, Spain; ^6^Immunogenetics and Histocompatibility Laboratory, Blood and Tissue Bank, Transfusional Medicine Group, Vall d'Hebron Research Institute, Barcelona, Spain

**Keywords:** Graves' disease, TSHR, tolerance, splicing isoforms, thymus, thyroid, next-generation sequencing

## Abstract

Graves' disease (GD) involves the presence of agonistic auto-antibodies against the thyrotropin receptor (TSHR), which are responsible for the clinical symptoms. While failure of TSHR tolerance is central to GD pathogenesis, the process leading to this failure remains poorly understood. Two mechanisms intimately linked to tolerance have been proposed to explain the association of SNPs located in *TSHR* intron 1 to GD: (1) differential alternative splicing in the thyroid; and (2) modulation of expression in the thymus. To elucidate the relative contribution to these two mechanisms to GD pathogenesis, we analyzed the level of full-length and ST4 and ST5 isoform expression in the thyroid (*n* = 49) and thymus (*n* = 39) glands, and the influence of intron 1-associated SNPs on such expression. The results show that: (1) the level of flTSHR and ST4 expression in the thymus was unexpectedly high (20% that of the thyroid); (2) while flTSHR is the predominant isoform, the levels are similar to ST4 (ratio flTSHR/ST4 = 1.34 in the thyroid and ratio flTSHR/ST4 in the thymus = 1.93); (3) next-generation sequencing confirmed the effect of the TSHR intron 1 polymorphism on TSHR expression in the thymus with a bias of 1.5 ± 0.2 overexpression of the protective allele in the thymus compared to the thyroid; (4) GD-associated intron 1 SNPs did not influence *TSHR* alternative splicing of ST4 and ST5 in the thyroid and thymus; and (5) three-color confocal imaging showed that TSHR is associated with both thymocytes, macrophages, and dendritic cells in the thymus. Our findings confirm the effect of intron 1 polymorphisms on thymic TSHR expression and we present evidence against an effect on the relative expression of isoforms. The high level of ST4 expression in the thymus and its distribution within the tissue suggest that this would most likely be the isoform that induces central tolerance to TSHR thus omitting most of the hinge and transmembrane portion. The lack of central tolerance to a large portion of TSHR may explain the relatively high frequency of autoimmunity related to TSHR and its clinical consequence, GD.

## Introduction

Graves' disease (GD) is a highly prevalent autoimmune disease characterized by the presence of agonistic auto-antibodies against the thyrotropin receptor (TSHR), which are responsible for hyperthyroidism and extrathyroidal manifestations (1, 2). Failure of tolerance to the TSHR is central to the pathogenesis of GD; however, our understanding of the process that leads to this failure remains incomplete.

The strong contribution of genetic factors to GD is best demonstrated in twin concordance studies that suggest that as much as 79% of the risk of developing GD is hereditary (3). During the past 25 years, classical genetic approaches and more recently, genome-wide association studies (GWAS), have identified several gene loci whose polymorphisms may contribute to GD susceptibility. Among the confirmed genes in the HLA region, *CTLA4* and *PTPN22* confer a higher risk; however, individual contributions, with the exception of HLA, are limited (4–6). The products of these genes participate in the regulation of the immune response and have been implicated in other autoimmune diseases but do not explain why the autoimmune response focuses on the thyroid gland. Common polymorphisms have also been found in the genes specifically expressed in the thyroid [e.g., the thyrotropin receptor [*TSHR*] or thyroglobulin [*TG*] (7, 8)]; however, only the association with *TSHR* has been repeatedly confirmed [reviewed in (4, 5, 9, 10)]. Since the loss of tolerance to TSHR is the central mechanism for GD pathogenesis, there is a great interest in understanding how these *TSHR* polymorphisms contribute to the failure of tolerance.

*TSHR* consists of 10 exons encoding a 764 amino acid protein of ~95 kDa that is converted to a 120 kDa protein following substantial glycosylation (11). The original TSHR peptide chain undergoes proteolytic cleavage, generating two subunits: (1) the A-subunit, encoded by exons 1–9, which constitutes the extracellular domain; and (2) the B-subunit, encoded by exon 10, which consists of a transmembrane region plus a 5 kDa C connecting peptide (12). Following the excision of the C peptide, the A- and B-subunits remain linked by disulfide bridges that can be subsequently reduced, and the A-subunit is partially shed, whereas B-subunit will remain anchored to the membrane (13, 14). Controversy remains regarding the persistence of single chain (uncleaved) TSHR on the surface of thyrocytes and to what extent subunit A is physiologically shed (15, 16).

The *TSHR* gene is expressed at medium to low levels in the thyroid (207 copies per million transcripts) and is extremely low in all other tissues (data from the GTEX portal, <0.3 transcripts per million in subcutaneous fat or 0.5 in the brain), except for AIRE-expressing orbital fibrocytes from GD patients with ophthalmic pathologies (17). As a reference, the classical housekeeping gene, *GAPDH*, has 813 transcripts per million in the thyroid.

**Figure 1:**
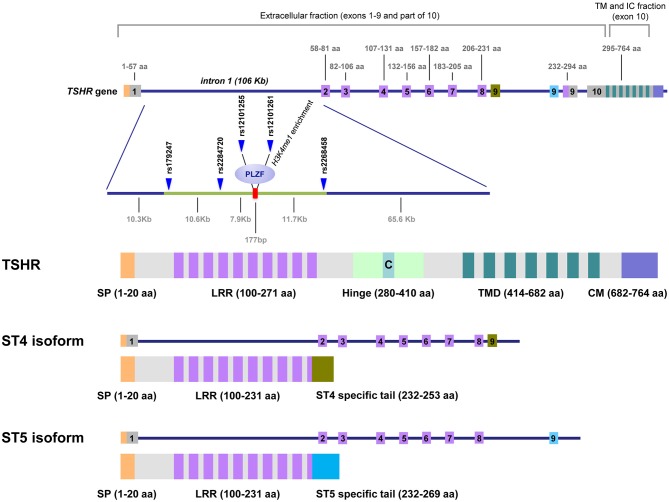
*TSHR* gene, GD-associated intron 1 polymorphisms and the predicted proteins expressed as cell-anchored proteins and as soluble forms. This figure shows the unique structure of TSHR. Intron 1 contains the GD-associated SNPs scattered on a region of ~30 kb at the 5′ end of intron 1. The numbers on top of the gene diagram correspond to the amino acids coded by each exon. The numbers on the bottom of the gene are the distances in kilobases (Kb). The primary associated SNPs are labeled in addition to the area of histone 3 lysine 4 monomethylated (H3K4me1), where the repressor factor, PLZF, was found to bind, reducing TSHR transcription. The exons used exclusively by the ST4 and ST5 isoforms are labeled in green and blue, respectively. The TSH holoreceptor (TSHR) and the two short isoforms (ST4 and ST5) are depicted, showing that the short isoforms contained less than half of the potential immunogenic regions of the receptor, including a large proportion of the extracellular domain, specially the hinge. C, C peptide; SP, signal peptide; LRR, Leucine Reach Repeats; TMD, transmembrane domain; CM, Cytoplasmic Motifs [This figure is based on Figure 1 and Figure 2 of manuscript (9). Reproduced with permission of Thieme editorial].

In addition to the full-length TSHR (flTSHR), five truncated TSHR transcripts have been reported in multiple studies, of which ST4 (1.3 kb) and ST5 (1.7 kb) are the most highly expressed (18–20). ST4 and ST5 share the first eight exons with the flTSHR but contain an additional ninth exon that is unique and different in ST4 and ST5, both of which lack exon 10. These unique exons are encoded in two different regions of intron 8, which are selected and retained in the mRNA of the corresponding alternatively spliced forms. If translated, both ST4 and ST5 would encode most of the leucine-rich repeats containing the TSH-binding extracellular region, but neither the hinge nor the transmembrane domain of the TSHR; therefore, is plausible that they are secreted (Figure 1).

Different studies have demonstrated a significant association between SNPs in the 40 kb region in the 5′ side of the large intron 1 (106 kb) of *TSHR* with GD [reviewed in (5)] (Figure 1). Two different but not mutually exclusive mechanisms have been postulated to explain this association. Brand et al. proposed that these SNPs influence mRNA splicing, which results in increased levels of the ST4 and ST5 transcripts encoding the short isoforms (21). The authors proposed that the short ST4 and ST5 variants would be translated into the putatively soluble TSHR forms, released into the circulation where they become available to antigen presenting cells in the periphery, where they may contribute to inducing an autoimmune response to the TSHR. However, it is unclear why these isoforms are immunogenic, rather than tolerogenic or ignored, in this hypothesis. In one experimental model, the soluble TSHR A subunit is more immunogenic than the membrane-bound TSHR (22); however, ST4 and ST5 differ from Subunit A in one exon and is difficult to predict their immunogenicity. Therefore, Brand et al. postulate the failure in peripheral tolerance as the crucial checkpoint for the development of GD.

The second proposed mechanism is based on our finding that the GD-associated SNPs in the intron 1 alleles modulate *TSHR* expression in the thymus. By measuring the levels of allele-specific *TSHR* mRNA in the thymus, we demonstrated that individuals carrying the protective genotype have higher levels of thymic *TSHR* mRNA compared to those with the disease-predisposing genotype. According to the well-established mechanisms of central tolerance, the expression of self-antigens in the thymus is required to develop a tolerant T cell repertoire (23, 24); a dose response relationship between the amount of a self-antigen in the thymus, and the frequency of T cells clones that can recognize it in the periphery, which has been demonstrated for the insulin gene in type 1 diabetes (25, 26) and is well-established in animal models (27).

Interestingly, Tomer et al. has recently provided some confirmatory evidence to our proposal, as they also found that intrathymic *TSHR* expression was lower in individuals homozygous for the disease-associated allele. In addition, these authors have shown that the effect of SNPs in intron 1 on the expression of *TSHR* may depend on the differential affinity of the allele for the PLZF transcription repressor factor. In turn, PLZF is regulated by IFN-α (28) and it is known that there is a strong IFN signature in the GD thyroid glands (29, 30). Moreover, IFN-α therapy can trigger thyroid autoimmunity when administered for hepatitis C virus infection (31). These authors propose that IFN-α can contribute to triggering GD by reducing *TSHR* expression in the thymus.

These two non-mutually exclusive mechanisms propose that the SNPs located in intron 1 participate in the regulation of *TSHR* expression through: (1) differential alternative splicing in the thyroid; and (2) modulation of expression levels in the thymus. To date, the effect of *TSHR* intron 1 polymorphisms on the differential expression of its isoforms (ST4 and ST5) has only been analyzed in the thyroid, in which the level of expression is relatively high compared with the thymus. However, although the level of TSHR expression and presumably its isoforms in the thymus is lower, it can be critical since it is in the thymus where the T lymphocyte repertoire is configured.

To better understand how the complex expression of *TSHR* in both the thymus and thyroid can modulate central and peripheral tolerance to TSHR, it is useful to precisely identify the levels of different isoform expression in these two tissues and analyze the influence of intron 1-associated SNPs on their expression. The results presented here constitute a detailed analysis of *TSHR* isoform expression in each tissue and provide insight into how TSHR tolerance may fail.

## Materials and Methods

### Patients and Samples

In this retrospective study, the diagnosis was made based on thyroid hormone levels, thyroid antibodies, including TSHR antibodies and ultrasound and scintiscan images, by experienced endocrinologists. Thyroid tissue was obtained from 49 patients (43 females, 7 males; age range: 15–71 years) recruited from the Endocrinology Clinics of Hospital Universitari Germans Trias i Pujol (HUGTiP) and Hospital Universitari Vall d'Hebron (HUVH), both of which are affiliated with the Universitat Autònoma de Barcelona (UAB) (Supplementary Table 1). The samples were processed as previously described (32).

Thymic tissue was obtained from 39 patients undergoing corrective cardiac surgery (31 pediatric and 8 adult patients; 15 females and 24 males; age range: 4 days−72 years), from the Departments of Heart Surgery of HUGTiP or HUVH. All samples were processed within 4 h of resection under sterile conditions, as previously described (33) (Supplementary Table 2).

Informed consent was obtained from all participants and the studies have been approved by the local institutional ethics review board of the participating institutions (ref PR AG-145/2011).

### DNA and RNA Isolation

Both genomic DNA (gDNA) and total RNA were isolated from the total thyroid and thymus tissue using standard methods (QIAamp DNA Mini QIAcube Kit and RNeasy Mini Kit, QIAGEN, Hilden, Germany). An additional step of DNase I treatment was applied to the RNA samples (RNase-free DNase set kit, QIAGEN). To check for contaminating gDNA in the RNA samples, 100 ng of the total RNA were subjected to 45 cycles of PCR using specific primers for a 309 nt non-transcribed region of the CTLA4 promoter. Only samples free of DNA were used for subsequent experiments.

### Genotyping TSHR Polymorphisms

DNA from the gland donors was genotyped for the two SNPs (rs179247 and rs12101255) known to be strongly associated with GD and for one non-associated SNP (rs2288495), by real-time PCR using TaqMan^®^ SNP genotyping assays (TaqMan^®^ SNP Genotyping Assay, Applied Biosystems, UK).

### Allele-Specific Quantification Using Massive Parallel Sequencing [Next-Generation Sequencing [NGS]]

To more precisely assess the effect of the rs179247 allele on the overall TSHR transcription, we used massive parallel sequencing (NGS). DNA-free RNA samples from the thyroid and thymus were obtained as described. To preserve the pre-mRNA in the complementary DNA (cDNA) synthesis step, retro-transcription of the RNA samples was performed using random primers (First Strand cDNA Synthesis Kit for RT-PCR [AMV], Roche, Basel, Switzerland). The specific primers were designed for the *TSHR* gene intron 1 region containing the rs179247 SNP (233 bp amplimer) (Supplementary Table 3).

#### Library Preparation, DNA Sequencing, SNP Calling, and Genotyping

Briefly, the target-specific primers were synthesized using CS1/CS2 tags (Fluidigm, San Francisco, CA) followed by a two-step PCR reaction. First, the region of interest was amplified with target-specific tagged primers, and second, the sample-specific barcode and adapters were introduced to generate the sequencing library (Fluidigm). A GeneAmp^®^ PCR System 9700 Thermal Cycler (Applied Biosystems, Foster City, CA) was used for the amplification. PCR products were pooled and purified using a MinElute Gel Extraction Kit (50) (Qiagen). The pool was run using the paired-end sequencing method on a MiSeq sequencing system (Illumina, San Diego, CA, USA) using the MiSeq reagent kit v2 (500 cycles) (Illumina). Finally, the FASTQ files were analyzed for the SNP rs179247 by read mapping and variant calling using the CLC Genomic Workbench software version 9.5.1 (CLC Bio, Qiagen, Aarhus, Denmark). A total of 19 thymus and 8 thyroid samples from heterozygous individuals for the SNP rs179247 were tested (Supplementary Table 4).

### Quantification of the Relative Gene Expression by qPCR

The level of gene expression for the full-length human *TSHR* mRNA (flTSHR) and two *TSHR* alternatively spliced transcripts (ST4 and ST5) were measured by qPCR using Taqman probes. DNA-free RNA samples from the thyroid and thymus were obtained as described above. The cDNA was synthesized using Oligo(dT) primers (First Strand cDNA Synthesis Kit for RT-PCR (AMV), Roche). To measure the level of flTSHR expression, we used a pre-designed TaqMan^®^ Gene Expression Assay that spans the *TSHR* exons 9 and 10 (assay ID: Hs01053841_m1). To measure the flTSHR, ST4, and ST5 transcripts, we used the primers and probes as described by Brand et al. (21) (Supplementary Table 5). When checking the sequences of the primers and probes, we realized that the ST5 probe sequence used by Brand et al. did not coincide with the ST5 consensus sequence by one nucleotide, and we modified the ST5 probe accordingly (Supplementary Table 5). Reactions were run on an Abi Prism^®^ 7900 HT instrument (Applied Biosystems) in triplicate and the CT average was used for further statistical analysis. The CV was always <15%. Normalization of the results was performed in accordance with the relative quantification 2-ΔΔCt method (34) with respect to the constitutive expression of the *GAPDH* gene. To display the results of the ST4 and ST5 isoform expression, we also used the ratio to flTSHR (35).

### TSHR Detection in Thymocytes and Thymic APCs by Indirect Immunofluorescence

To investigate how TSHR is presented in the thymus, we stained thymic 5 μm cryostat sections with monoclonal antibodies (mAbs) specific for TSHR (clone 49, Thermofisher, Waltham, MA, USA), CD68 as a macrophage marker (clone Y1/82A Biologend, San Diego, CA, USA), and CD11c as a dendritic cell marker (clone CBR-p150/4G1, Thermofisher). To avoid cross-reactivity, the Ig isotype or species-specific secondary labeled anti-sera were used. For each protocol, the controls included sections incubated with the conjugated secondary anti-sera without the corresponding primary antibody to assess the background. Sections in which each of the primary antibodies in the protocol were omitted to detect any possible cross-reactions. Samples from five different glands (1 male and four female donors; age range: 10 months to 3 years and 7 months) were used. The sections were first examined under a UV photomicroscope equipped with the adequate filters and selected sections were examined under a confocal microscope (FV1000, Olympus Corporation, Tokyo, Japan).

### Statistical Analysis

Descriptive data were presented as the mean ± standard deviation (SD) or median and semi interquartile range (SIR). The data were analyzed using a non-parametric test, U-Mann Whitney test, or Kruskal-Wallis test with a multiple comparisons correction (Dunn's test), except for the analysis of the NGS data, which followed a normal distribution (*t*-test). The accepted *p*-value is <0.05. We used GraphPad Prism 5.0 program (GraphPad Software, La Jolla CA) for the statistical analysis and figure generation.

## Results

### Analysis of *TSHR* Transcripts in the Thymus and Thyroid

**Figure 2:**
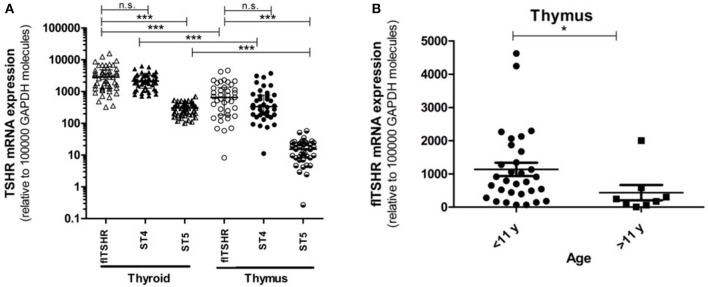
Gene expression of the *TSHR* isoforms in the thymus and thyroid. **(A)** mRNA expression of the full-length TSHR (flTSHR) and its isoforms (ST4 and ST5) in the thymus and thyroid. The relative level of mRNA expression was measured using quantitative PCR of the cDNA from 49 thyroid and 39 thymus samples. Each sample was tested in triplicate and the standard deviation was always <15% of the mean value. Values were normalized to *GAPDH* expression using the comparative CT method and expressed as copies per 100,000 copies of *GAPDH*. Each point represents the mean value of triplicate results from one sample. The median ± IQR of each group is shown. ^***^p < 0.0001, Kruskal Wallis test with a Dunn's multiple comparison test. **(B)** The relative mRNA expression of flTSHR in the thymus samples was stratified into pediatric (0–11 years; *n* = 31) and adults (40–80 years; *n* = 8) samples. The mean ± SEM of each group is shown. ^*^p < 0.05 using a *t*-test for independent samples.

We analyzed the relative expression of full-length TSHR (flTSHR) transcripts by qPCR and its two major alternatively spliced transcripts (ST4 and ST5) from the cDNA of 39 and 49 samples collected from the thymus and thyroid tissue, respectively. The level of flTSHR expression in the thymus was higher than expected, which was ~20% of the expression observed in the thyroid (median thymus: 651 ± 580 vs. median thyroid: 2860 ± 1792) (Figure 2A). Although these expression levels are similar to those mentioned by Kim van der Weerd (36), they are higher than the expression levels reported in several databases [e.g., BioGps (37) or EMBL-EBI Expression Atlas [https://www.ebi.ac.uk/gxa]]. Recently, this relatively high level of ST4 expression in the thymus was also reported by Latif et al., who designated it TSHR v1.3 (16).

Age was found to have an effect on *TSHR* expression in the thymus. The average level of flTSHR expression in donors aged 0 to 11 years-old was 1,135 ± 1,110 and from donors ages 40 to 80 years old was 436 ± 657 (*p* < 0.05) (Figure 2B). Since our thymus samples were from relatively young patients, this may explain the discrepancy with levels recorded in the databases.

**Figure 3:**
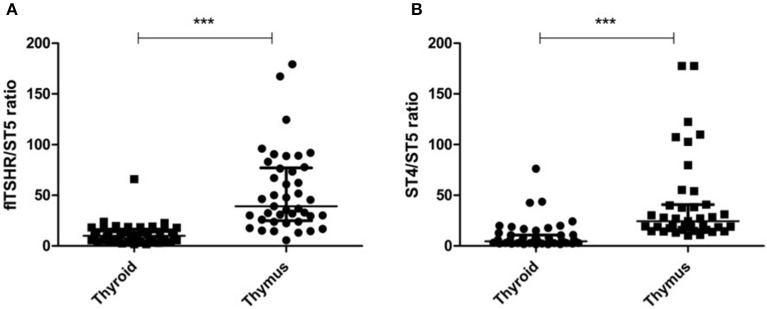
Proportionally lower expression of the TSHR ST5 isoform in the thymus compared with the thyroid. The expression ratios of ST5 with flTSHR **(A)** and ST4 **(B)** in the thymus and thyroid were calculated. Each point represents the mean value of triplicate results from one sample. The median ± IQR of each group is shown. ^***^p < 0.0001 using a *t*-test for independent samples.

It is of interest that the relative levels of flTSHR and ST4 expression were not as different as expected in the thymus and thyroid and they were higher for flTSHR than for ST4 (thyroid flTSHR 2,860 ± 1,792, ST4 2,135 ± 961, ratio flTSHR/ST4 = 1.34; thymus flTSHR 651 ± 580, ST4 337 ± 299, ratio flTSHR/ST4 = 1.93). In contrast, the level of isoform ST5 expression was substantially lower in both tissues (thyroid ST5 303 ± 104 and thymus ST5 15 ± 8). Interestingly, the level of ST5 expression was proportionally much lower in the thymus compared with the thyroid (ST5 represents 6% compared to 1.5% of the total transcripts (flTSHR+ ST4 + ST5) in the thyroid and thymus, respectively) (Figure 2A). This difference is also reflected when comparing the ratios of ST5 expression with flTSHR or ST4 in both tissues (thyroid flTSHR/ST5 = 9.86 vs. thymus flTSHR/ST5 = 39.01; *p* < 0.0001) (Figure 3A) (thyroid ST4/ST5 = 4.5 vs. thymus ST4/ST5 = 24.3; *p* < 0.0001) (Figure 3B).

The above results indicate that substantial soluble TSHR likely exists within the thymus, which is primarily derived from the transcription of ST4. Thus, this expression may have implications for establishing central TSHR tolerance.

### NGS Confirms the Thymus-Specific Influence of *TSHR* Expression by GD-Associated SNPs

We previously demonstrated that the risk allele, rs179247 (one of the main *TSHR* GD-associated SNPs), determined lower *TSHR* expression in the thymus but not in the thyroid (38). However, since we used a semiquantitative technique (allele-specific transcript quantification by qPCR using FRET probes), we decided to apply massive parallel sequencing, which is a more reliable quantitative technique, for the comparison of rs179247 *TSHR* allele expression in heterozygous individuals. It should be emphasized that since rs179247 is located in intron 1, we took care in obtaining the cDNA from the pre-mRNA (immature RNA before the splicing process). The designed primers do not discriminate between cDNA and gDNA and consequently, the contaminating gDNA must be absent to obtain reliable results.

**Figure 4:**
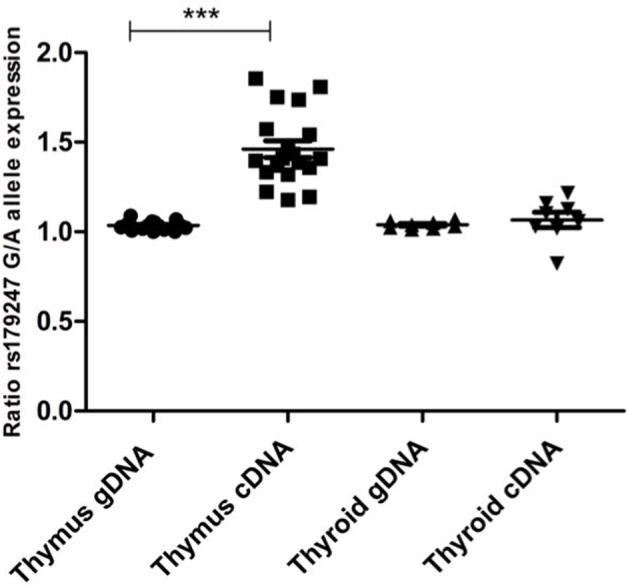
Preferential transcription of the rs179247 GD protective allele in the thymus. We used massive parallel sequencing to determine the relative abundance of *TSHR* alleles in 19 thymus and 8 thyroid cDNA samples from individuals heterozygous for the GD-associated SNP, rs179247. Specific primers were designed for the intron 1 region of the *TSHR* gene containing the analyzed SNP. Allele-specific quantification was measured in the gDNA, as a control for equal biallelic representation, and the cDNA in all samples. Each point represents the ratio between the G (protecting) and the A (predisposing) allele reads. ^***^p < 0.001 using a Kruskal Wallis test with Dunn's multiple comparison test.

Allele-specific quantification was measured in the gDNA and the cDNA from 19 thymus and 8 thyroid samples from heterozygous donors (previously genotyped for rs179247). We obtained good coverage, with a mean depth in the gDNA samples of 27,000 × (i.e., 27,000 sequences or “reads” interrogating the rs179247 position) and 9,000 × in the cDNA samples, with no significant differences between the thymus and thyroid samples (Table 1). The results showed that the ratio between the G (protecting) and the A (predisposing) alleles for gDNA was always 1 in both the thyroid and thymus, reflecting the existence of the same number of maternal and paternal DNA copies in each cell (Figure 4; Table 1). For cDNA, the percentage of reads corresponding to the G allele in the thyroid was ~50% (range: 45.1–54.8%) with a G/A allele ratio of 1 ± 0.1 SD. In contrast, the G/A allele ratio in the thymus was 1.5 ± 0.2 SD, with a mean of 59.1% (ranging: 54.1–65%). These findings indicate that there is unbalanced allele transcription only in the thymus (reflecting a tissue-specific effect) and demonstrates that the protective *TSHR* G allele is preferentially transcribed.

**Table 1:** Allele-specific transcript quantification by NGS.

	**Sample type**	**Depth of coverage^a^**	**Balance^b^**	**Counts (G allele)**	**Frequency (G allele) %**
**THYROID**					
102	gDNA	29548x	0.49	15,054	50.95
	**cDNA**	**9305x**	**0.49**	**4,926**	**52.94**
218	gDNA	25800x	0.49	13,283	51.48
	**cDNA**	**5006x**	**0.49**	**2,746**	**54.85**
355	gDNA	15027x	0.49	7,771	51.71
	**cDNA**	**9200x**	**0.50**	**4,940**	**53.70**
381	gDNA	25044x	0.49	12,633	50.44
	**cDNA**	**23579x**	**0.49**	**12,361**	**52.42**
409	gDNA	25049x	0.50	12,726	50.80
	**cDNA**	**14221x**	**0.49**	**7,211**	**50.71**
430	gDNA	29863x	0.49	15,038	50.36
	**cDNA**	**1693x**	**0.49**	**764**	**45.13**
452	gDNA	32564x	0.49	16,484	50.62
	**cDNA**	**1738x**	**0.50**	**877**	**50.46**
92	gDNA	20911x	0.49	10,717	51.25
	**cDNA**	**13439x**	**0.49**	**6,916**	**51.46**
**THYMUS**					
101	gDNA	28664	0.49	14,453	50.42
	**cDNA**	**6933**	**0.49**	**3,960**	**57.12**
117	gDNA	35762	0.49	18,282	51.12
	**cDNA**	**5912**	**0.49**	**3,764**	**63.67**
119	gDNA	24245	0.49	12,165	50.18
	**cDNA**	**4570**	**0.49**	**2,663**	**58.27**
126	gDNA	28552	0.50	14,280	50.01
	**cDNA**	**4510**	**0.49**	**2,481**	**55.01**
145	gDNA	33290	0.50	16,932	50.86
	**cDNA**	**17575**	**0.49**	**9,996**	**56.88**
159	gDNA	25178	0.50	12,664	50.30
	**cDNA**	**6616**	**0.49**	**4,299**	**64.98**
16	gDNA	22447	0.49	11,221	49.99
	**cDNA**	**13429**	**0.49**	**8,649**	**64.41**
171	gDNA	30800	0.49	15,640	50.78
	**cDNA**	**4138**	**0.49**	**2,438**	**58.92**
188	gDNA	29066	0.49	14,720	50.64
	**cDNA**	**342**	**0.48**	**185**	**54.09**
195	gDNA	31457	0.49	16,255	51.67
	**cDNA**	**3656**	**0.50**	**2,235**	**61.13**
197	gDNA	31543	0.49	16,169	51.26
	**cDNA**	**8058**	**0.49**	**4,385**	**54.42**
225	gDNA	26017	0.49	13,152	50.55
	**cDNA**	**18406**	**0.49**	**11,164**	**60.65**
229	gDNA	31049	0.50	15,955	51.39
	**cDNA**	**7373**	**0.50**	**4,392**	**59.57**
235	gDNA	31949	0.49	16,325	51.10
	**cDNA**	**14662**	**0.49**	**8,516**	**58.08**
263	gDNA	33124	0.49	16,852	50.88
	**cDNA**	**16402**	**0.49**	**9,478**	**57.79**
28	gDNA	27355	0.49	13,931	50.93
	**cDNA**	**6143**	**0.49**	**3,593**	**58.49**
38	gDNA	4047	0.49	2,110	52.14
	**cDNA**	**6701**	**0.50**	**4,253**	**63.47**
4	gDNA	25273	0.49	12,879	50.96
	**cDNA**	**16769**	**0.49**	**9,802**	**58.45**
72	gDNA	31112	0.49	15,957	51.29
	**cDNA**	**7426**	**0.49**	**4,277**	**57.59**

*Results of cDNA samples are indicated in bold*.

a *The number of unique reads (i.e., sequences) which interrogate the position of rs179247*.

b *Balance between reads from the forward or reverse primer. A value of 0.5 indicates that half of the reads come from each primer (i.e., there is no strand bias)*.

### GD-Associated Intron 1 SNPs Do Not Influence Alternative *TSHR* Splicing in the Thyroid and Thymus

**Figure 5:**
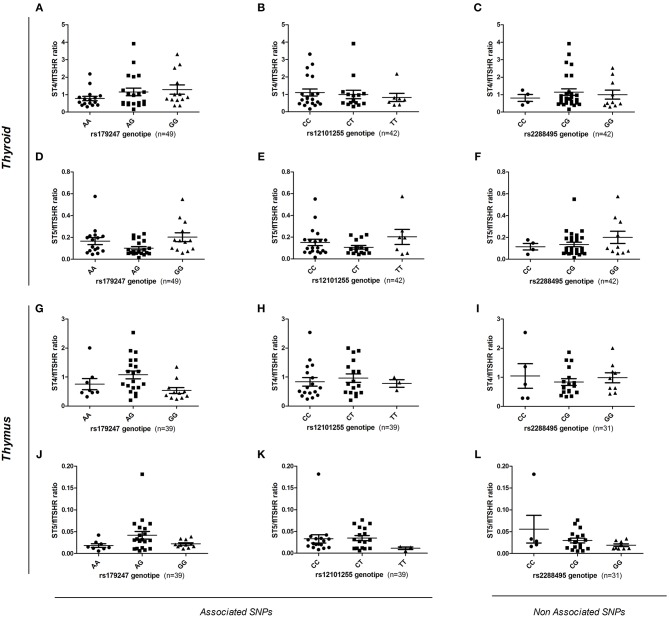
Effect of GD-associated SNPs (rs179247 and rs12101255) on the expression of TSHR alternatively spliced isoforms in human thyroid and thymus samples. The relative level of flTSHR, ST4, and ST5 expression was measured by qPCR and normalized to the level of GAPDH expression. The ratio of each short ST4 and ST5 transcript to the flTSHR was calculated for each individual and the data were grouped by SNP genotype. **(A–F)** The relative ST4 and ST5 expression in the thyroid is presented. **(G–L)** Relative level of ST4 and ST5 expression in the thymus is presented. A non-GD-associated SNP (rs2288495) was also included as a control. Each point represents the mean value of triplicate results from one sample. The mean ± SEM of each group is shown. A Kruskal-Wallis with Dunn's multiple comparison test was used.

To assess the effect of intron 1 GD-associated SNPs on the differential transcription of alternatively spliced *TSHR* isoforms, gDNA samples from donors (*n* = 88, 49 thyroid and 39 thymus donors) were genotyped for the rs179247 and rs12101255 SNPs associated with GD, and for the control SNP, rs2288495 (located in the 3' UTR of *TSHR*, not in linkage disequilibrium with rs179247; *r* = 0.05) (21, 38). The relative level of flTSHR, ST4, and ST5 expression was measured in the thymus and thyroid by qPCR and normalized to the level of *GAPDH* expression. The ratio of each of the truncated ST4 and ST5 transcripts to flTSHR was calculated for each individual and the data were grouped by SNP genotype (Figure 5).

In the thyroid samples, there was no overall significant effect of the genotypes on ST4 or ST5 expression, either normalized to GAPDH or expressed as a ratio to flTSHR (Figures 5A–F). Specifically, there were no statistically significant differences between the protective (GG for rs179247 and CC for rs12101255) and the predisposing genotypes (AA for rs179247 and TT for rs12101255). These results differ from those reported by Brand et al. in their original publication (21).

In the analysis of the relative expression of the ST4 and ST5 transcripts in the thymic samples, there is no overall significant effect of the genotypes for ST4 or ST5 expression relative to flTSHR (Figures 5G–L). There are no statistically significant differences between the protective and the predisposing genotypes. In both the thymus and thyroid, the control SNP was not associated with GD (rs2288495), but was also not significantly different.

In addition to the codominant and the most likely recessive models considered above, we have also considered the dominant model for the rs179247G and rs21201255C alleles; however, no statistical differences were found for either of these models (Supplementary Figure 1).

### TSHR Is Associated With Both Thymocytes, Macrophages, and Dendritic Cells in the Thymus

**Figure 6:**
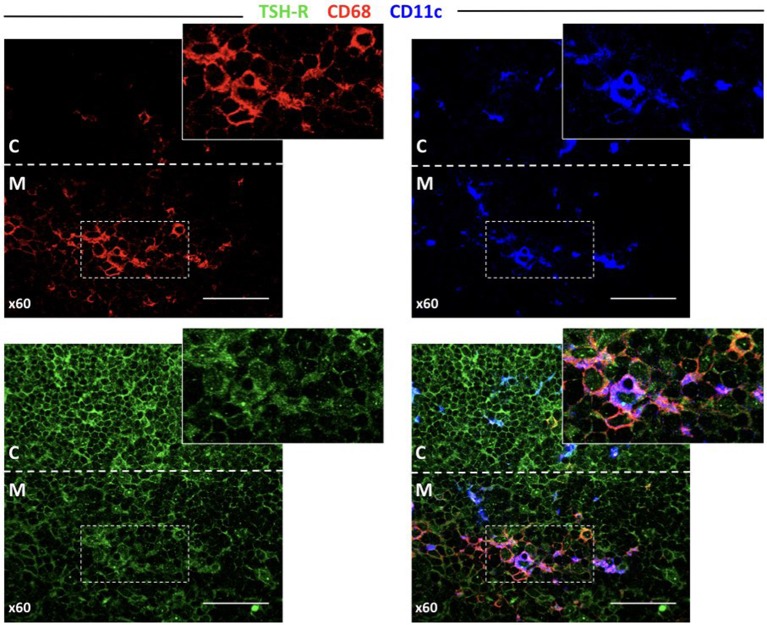
TSHR protein expression in the thymus detected by immunofluorescence. Abundant cells in the cortex that can be morphologically identified as thymocytes and other larger cells (left bottom panel) are positive for TSHR visualized in the green channel. The bottom-right pictures combine the macrophage marker, CD68, and the dendritic cell marker, CD11c, reveal some orange-yellow cells that correspond to macrophages that contain TSHR. There are also some violet cells (probably DCs), for which is more difficult to identify TSHR staining but some cytoplasmic light blue fluorescence (from the overlapping of dark blue and green), is observed. The arrow points to a macrophage visible in all micrographs except the CD11c staining, and the arrowhead points to a dendritic cell visible in all micrographs except in the CD68 staining.

It would be extremely interesting to elucidate how TSHR and its isoforms are processed and presented in the thymus, as this should determine the level of central tolerance and more specifically, which epitopes escape this central tolerance. Since there are no TSHR isoform-specific antibodies available, after testing several reagents, we finally selected a mAb that resulted in clear staining in the thyroid and low background in the lymphoid tissue (Supplementary Figure 2). As seen in Figure 6, the thymocytes located in the inner cortical areas of the thymus are clearly stained for TSHR. Interestingly, some positive dots were observed, indicative of small quantities of TSHR associated with both CD68^+^ macrophages and CD11c^+^ dendritic cells.

## Discussion

In this article, we report three significant findings: (1) relatively high expression of the short ST4 and ST5 *TSHR* transcripts was observed in the thymus, which may have implications for the establishment of central TSHR tolerance; (2) massive parallel sequencing was used to definitively correlate the differential effect of GD-associated rs179247 SNP alleles on *TSHR* transcription in the thymus but not in the thyroid; and (3) there was an absence of an effect of GD-associated SNPs (rs179247 and rs12101255) in modulating mRNA splicing in the thyroid and thymus, resulting in similar levels of ST4 and ST5 transcripts between samples from individuals homozygous for the risk or protective genotype.

This is the first study to simultaneously address the two mechanisms proposed to explain the functional role of *TSHR* intron 1 GD-associated SNPs. The mechanism, which involves defective central tolerance, was initially proposed by our group. In our previous study, we showed that individuals with the rs179247 GD-protective genotype displayed higher levels of thymic *TSHR* expression than those with the disease-associated genotype (38). This finding has been also later reported by Yaron Tomer's group (28). In both studies, the results were obtained measuring gene expression by qPCR in genotyped individuals; however, since this approach is vulnerable to individual confounding factors that can influence the results (e.g., individual genetic background, age, and sex differences), we used an allele-specific quantification (ASQ) method to quantify the contribution of each allele (risk vs. protective) in heterozygous individuals. This ASQ method was a qPCR-based semiquantitative technique and confirmed higher levels of protective allele expression in the thymus (38). In the present study, we aimed to definitely corroborate these results using a more robust quantitative technique of massive parallel sequencing (NGS). The high on-target coverage that was obtained (>9,000 reads in cDNA samples) allowed us to precisely quantify the number of transcripts expressed from each allele in heterozygous individuals. We confirmed the unbalanced tissue-specific transcription of the two alleles with the observation that the GD-protective allele was expressed 1.5-fold more on average, compared to the GD-predisposing allele in the thymus, but not in the thyroid. Although it remains unclear how these moderate differences in the expression of a self-antigen in the thymus have such an important effect on establishing the level of central tolerance, they may be related to the limited window of opportunity that maturing thymocytes have to interact with tissue-restricted antigens in the thymus (39). The magnitude of this imbalance here reported by NGS is in the same order of magnitude of that detected by ourselves using ASQ by qPCR (38), and of that reported by Pugliese et al. and Vafiadis et al. for the insulin gene in the first publications describing this phenomenon (25, 40).

The second mechanism used to explain the functional role of *TSHR* intron 1 GD-associated SNPs was proposed by Brand et al. and highlighted the differential regulation of mRNA splicing by these SNPs. The authors showed that GD-risk alleles of intron 1 SNPs (rs179247 and rs12101255) were associated with a relative increase in ST4 and ST5 expression, which would result in a higher production of variants encoding putatively more antigenic soluble TSHR isoforms (21). In our study, when we attempted to replicate this experiment, rs179247 and rs12101255 did not affect the transcription of the ST4 and ST5 isoforms in the thyroid or thymus. Although these results differ with those reported by Brand et al. for an unknown reason, this discrepancy may be the result of our study involving a much larger series of thyroid samples. We used 49 thyroid samples compared with the 12 samples used by Brand et al. In their study, when the 12 samples were divided by genotype, the result was small groups consisting of only three to five samples each (one of which had a high dispersion of the values), which the authors stated required further confirmation (21). Additionally, the ST5 probe sequence described in Brand et al. had an incorrect nucleotide when compared with the current reference genome. Furthermore, it is difficult to predict if this mismatch had an effect on ST5 quantification.

We believe that our results are robust due to the use of highly reliable and quantitative methods (NGS for ASQ and probe-specific qPCR for gene expression). The age, clinical conditions, and presence of thyroid autoantibodies in the donors had no effect on the results, and they were excluded as confounding factors (data not shown). Therefore, we can conclude that *TSHR* intron 1 GD-linked SNPs are associated with different levels of thymic *TSHR* expression, but not with differential mRNA splicing.

Perhaps the most striking finding in the present study is the demonstration of high levels of short *TSHR* transcript expression, especially ST4, in the thymus [recently confirmed by Latif et al. (16)]. This is relevant if we consider the peculiar distribution of *TSHR* expression within the thymus. Different from most other restricted tissue antigens (RTAs), while the *TSHR* receptor is only minimally expressed by medullary thymic epithelial cells (mTECs), it is expressed at relatively high levels by double positive thymocytes themselves, for which it appears to play a role in maturation and differentiation (9, 36, 38, 41). Therefore, it should be assumed that there are two sources of TSHR protein in the thymus: (1) the TSHR anchored in the thymocyte membrane and the soluble ST4 and ST5 isoforms generated by alternative splicing. We and others consider that the ST4 and ST5 isoforms are translated (ST4 and ST5 mRNA have all the features of mature transcripts), even if no experimental evidence is available. According to the transcript levels, soluble short TSHR isoforms may be present at levels comparable to that of membrane-anchored TSHR. It would be important to determine which of these two forms (soluble vs. membrane-anchored) play the main role in the establishment of central tolerance to TSHR. It is difficult to answer to this question with certainty given the present data and reagents. As previously shown by van der Weerd (36) and ourselves (41), as well as by our IFL experiments in this paper, TSHR is mainly expressed in double positive thymocytes; however, low levels are also observed in both macrophages and dendritic cells. There are currently no reagents that can be used to identify the isoforms detected by IFLs in these APCs. Massive quantities of double positive thymocytes die by apoptosis in the thymus cortical compartment. It is known that they are disposed by macrophages that ingest apoptotic cells. Importantly, macrophages are much less efficient than mTEC or thymic dendritic cells at inducing tolerance to self-autoantigens, which should apply to thymocyte antigens (42, 43). In contrast, soluble TSHR isoforms are likely engaged by thymic dendritic cells, which are highly efficient at inducing negative selection. If central tolerance to TSHR is predominantly dependent on the two soluble ST4 and ST5 isoforms, the C terminal segment consisting of 533 aa out of the 764 aa of the full length TSHR molecule would not be subjected to the tolerization process. In GD, although the pathogenic stimulating antibodies are directed at the N terminal LRR in the ectodomain, tolerance is ultimately dependent on T cells. Thus, B cells that recognize the ectodomain may receive help from T cells that recognize epitopes in the long C terminal stretch of TSHR, to which tolerance is incomplete. This is an important issue because a demonstrated mechanism of central tolerance failure is the expression of different protein isoforms in both the thymus and periphery. This was first demonstrated by Klein and Kyewski in experimental acute encephalomyelitis induced by the myelin proteolipid protein in SJL/J mice (44). Since this original description, similar results have also been demonstrated for the islet antigen, I-A2 (45), and is also used to explain autoantibodies to post-translationally modified proteins (e.g., citrullinated peptides in rheumatoid arthritis) (46). In addition, the role of TSHR expression in the thymus for protection against the development of pathogenic anti-TSHR antibodies has recently been demonstrated in a mouse model (47). Therefore, we propose that a crucial mechanism for the failure of TSHR tolerance is the lack of presentation of the full-length TSHR molecule by tolerogenic APCs in the thymic medulla.

Collectively, the results of the present study support the functional role of *TSHR* intron 1 GD-associated SNPs in modulating central tolerance through influencing the intrathymic expression of *TSHR*. In addition, these findings provide a novel explanation as to why the loss of tolerance to TSHR occurs with relatively high frequency (i.e., differential expression of isoforms in the thymus vs. thyroid). Furthermore, a deeper analysis of the differential isoform expression (i.e., measurements of the corresponding proteins, the presence of TSHR peptides in the thymic ligandome) and epitope specificity of TSHR-specific autoreactive T cells should be assessed in future studies.

## Data Availability

This manuscript contains previously unpublished data. The name of the repository and accession number(s) are not available.

## Ethics Statement

This study was carried out in accordance with the recommendations of local institutional ethics review board of the participating institutions, with written informed consent from all subjects. All subjects gave written informed consent in accordance with the Declaration of Helsinki. The protocol was approved by Vall d'Hebron Hospital (HUVH) Research Ethics Comittee (Approval reference PR AG-145/2011).

## Author Contributions

AM-S collected the samples, performed the qPCR experiments, analyzed the data, and wrote part of the manuscript. AS-S contributed to the collection and processing of the samples, as well as the qPCR experiments. FR and EE performed the ASQ NGS experiments. DÁ-S performed the immunofluorescence experiments. AL-M and OG reviewed the clinical and surgical data in patient records. RC and RP-B were responsible for designing the study, checking the experimental protocols and results and writing the manuscript, and approving the final draft.

### Conflict of Interest Statement

The authors declare that the research was conducted in the absence of any commercial or financial relationships that could be construed as a potential conflict of interest.
