# Supplementary material for: Regulation of TSHR Expression in the Thyroid and Thymus May Contribute to TSHR Tolerance Failure in Graves' Disease Patients via Two Distinct Mechanisms
Source: Front Immunol. 2019 Jul 18;10:1695. doi: 10.3389/fimmu.2019.01695 (PMC6657650; doi:10.3389/fimmu.2019.01695)
Supplement: Supplementary file 1 [file Data_Sheet_1.docx]

**Supplementary table 1**. Thyroid samples.

| Sample | rs179247 | rs2288495 | rs1210225 | Sex | Age | Pathology | Tissue |
| --- | --- | --- | --- | --- | --- | --- | --- |
| TB 057 | A/G | C/G | C/C | M | 53 | GD | Thyroid |
| TB 081 | G/G | G/G | C/C | F | 32 | GD | Thyroid |
| TB 092 | A/G | C/G | C/T | F | 42 | GD | Thyroid |
| TB 102 | A/G | C/G | C/T | F | 38 | GD | Thyroid |
| TB 134 | G/G | C/G | C/C | F | 22 | GD | Thyroid |
| TB 157 | A/G | C/G | C/T | F | 32 | GD | Thyroid |
| TB 183 | A/G | C/G | C/C | F | 34 | GD | Thyroid |
| TB 186 | G/G | C/G | C/C | F | 41 | GD | Thyroid |
| TB 209 | A/G |  | C/C | M | 50 | NORMAL | Thyroid |
| TB 218 | A/G | C/G |  | F | 38 | MNG | Thyroid |
| TB 226 | G/G | C/C | C/C | F | 53 | MNG | Thyroid |
| TB 277 | G/G | C/G | C/C | F | 51 | GD | Thyroid |
| TB 298 | A/G | C/G | C/T | M | 43 | MNG | Thyroid |
| TB 307 | A/G | C/G | C/T | F | 45 | MNG | Thyroid |
| TB 308 | G/G | G/G | C/C | F | 25 | GD | Thyroid |
| TB 323 | G/G |  | C/C | F | 39 | MNG | Thyroid |
| TB 335 | G/G |  | C/C | F | 48 | MNG | Thyroid |
| TB 339 | G/G |  | C/C | F | 43 | MNG | Thyroid |
| TB 355 | A/G | C/G | C/C | F | 49 | MNG | Thyroid |
| TB 363 | G/G | C/G | C/C | M | 71 | MNG | Thyroid |
| TB 366 | G/G | G/G |  | F | 36 | MNG | Thyroid |
| TB 368 | A/G | C/G | C/C | F | 54 | MNG | Thyroid |
| TB 379 | G/G | C/G | C/C | F | 56 | MNG | Thyroid |
| TB 381 | A/G | C/G | C/T | F | 45 | GD | Thyroid |
| TB 390 | A/A | G/G | C/T | F | 35 | GD | Thyroid |
| TB 391 | A/A | G/G | C/C | M | 38 | GD | Thyroid |
| TB 394 | A/G | C/C |  | F | 49 | GD | Thyroid |
| TB 395 | A/A | G/G | C/T | F | 41 | MNG | Thyroid |
| TB 404 | A/G | C/G | C/T | F | 55 | MNG | Thyroid |
| TB 406 | A/A |  |  | F | 37 | MNG | Thyroid |
| TB 409 | A/G | C/G | C/T | F | 62 | MNG | Thyroid |
| TB 412 | A/A | G/G | C/C | F | 25 | GD | Thyroid |
| TB 413 | A/A | G/G | T/T | F | 40 | GD | Thyroid |
| TB 416 | A/A | G/G | T/T | F | 20 | GD | Thyroid |
| TB 420 | G/G | C/G |  | F | 45 | MNG | Thyroid |
| TB 421 | A/A | C/G | C/T | F | 31 | GD | Thyroid |
| TB 423 | A/A | C/C | T/T | F | 33 | GD | Thyroid |
| TB 426 | A/G | C/G | C/T | M | 40 | GD | Thyroid |
| TB 429 | A/A | C/C | T/T | F | 28 | GD | Thyroid |
| TB 430 | A/G | C/G | C/C | F | 30 | GD | Thyroid |
| TB 431 | A/G | C/G | C/T | F | 32 | GD | Thyroid |
| TB 439 | A/A |  |  | F | 70 | MNG | Thyroid |
| TB 442 | A/G | C/G | C/T | F | 24 | GD | Thyroid |
| TB 443 | A/A | G/G | C/T | F | 50 | GD | Thyroid |
| TB 446 | A/A | C/G | T/T | F | 24 | GD | Thyroid |
| TB 450 | A/A | C/G | T/T | M | 24 | GD | Thyroid |
| TB 451 | A/A |  |  | F | 33 | MNG | Thyroid |
| TB 452 | A/G | C/G | C/C | F | 23 | GD | Thyroid |
| TB 464 | A/A | C/G | T/T | F | 15 | GD | Thyroid |

GD: Graves-Basedow disease; MNG: Multinodular goiter

**Supplementary table 2**. Thymus samples.

| Sample | rs179247 | rs2288495 | rs1210225 | Sex | Age | Tissue |
| --- | --- | --- | --- | --- | --- | --- |
| TMB 060 | G/G | C/C | C/C | M | 3m | Thymus |
| TMB 072 | A/G | C/G | C/T | M | 3m | Thymus |
| TMB 088 | A/A | G/G | C/T | M | 4y6m | Thymus |
| TMB 093 | A/A | C/G | T/T | M | 5m | Thymus |
| TMB 097 | G/G | C/G | C/C | M | 1m | Thymus |
| TMB 099 | A/G | C/C | C/C | F | 62y | Thymus |
| TMB 100 | A/A | C/G | C/T | F | 6y | Thymus |
| TMB 101 | A/G | C/G | C/C | M | 7m | Thymus |
| TMB 109 | A/A | C/G | T/T | M | 4d | Thymus |
| TMB 113 | A/G | C/G | C/T | M | 47y | Thymus |
| TMB 117 | A/G | C/G | C/T | M | 11y | Thymus |
| TMB 119 | A/G | C/G | C/T | M | 8m | Thymus |
| TMB 125 | A/A | G/G | C/T | M | 11d | Thymus |
| TMB 126 | A/G | C/G | C/T | F | 1y5m | Thymus |
| TMB 128 | A/G | G/G | C/T | F | 50y | Thymus |
| TMB 133 | A/G | C/G | C/C | M | 51y | Thymus |
| TMB 135 | A/G | C/G | C/C | M | 44y | Thymus |
| TMB 137 | G/G | C/G | C/T | F | 3m | Thymus |
| TMB 139 | G/G | G/G | C/C | F | 1y7m | Thymus |
| TMB 142 | G/G | G/G | C/C | M | 3m | Thymus |
| TMB 175 | A/A | C/G | T/T | M | 3m | Thymus |
| TMB 177 | A/G | C/C | C/C | F | 53y | Thymus |
| TMB 178 | A/G | G/G | C/C | F | 72y | Thymus |
| TMB 186 | A/G | G/G | C/T | F | 50y | Thymus |
| TMB 188 | A/G | G/G | C/C | M | 9d | Thymus |
| TMB 195 | A/G | G/G | C/T | M | 11m | Thymus |
| TMB 197 | A/G | C/G | C/T | F | 7m | Thymus |
| TMB 203 | G/G |  | C/C | F | 1y10m | Thymus |
| TMB 218 | G/G |  | C/C | M | 1y4m | Thymus |
| TMB 225 | A/G |  | C/T | F | 3y5m | Thymus |
| TMB 229 | A/G |  | C/T | F | 7m | Thymus |
| TMB 232 | G/G |  | C/C | M | 8y5m | Thymus |
| TMB 247 | A/A |  | C/T | M | 5d | Thymus |
| TMB 261 | A/A |  |  | M | 3y5m | Thymus |
| TMB 263 | A/G |  | C/T | F | 4y5m | Thymus |

**Supplementary table 3**. Primers for allele-specific transcript quantification by NGS.

| rs179247 Primer forward (23 nt) | 5’-CCATGCCTGTGTGTTCTCATAAT-3’ |
| --- | --- |
| rs179247 Primer reverse (22nt) | 5’-GCCATCTCAAAGCCCACTAAAG-3’ |
| Adaptador (CS1tag, forward) | 5’-ACACTGACGACATGGTTCTACA-3’ |
| Adaptador (CS2tag, reverse) | 5’-TACGGTAGCAGAGACTTGGTCT-3’ |

**Supplementary table 4**. Thymus and thyroid samples included in NGS.

| Sample | Tissue | Sex | Age | Diagnostic |
| --- | --- | --- | --- | --- |
| TB092 | Thyroid | F | 42y | Graves Disease |
| TB102 | Thyroid | F | 38y | Graves Disease |
| TB218 | Thyroid | F | 38y | Multinodular goiter |
| TB355 | Thyroid | F | 49y | Multinodular goiter |
| TB381 | Thyroid | F | 45y | Graves Disease |
| TB409 | Thyroid | F | 62y | Multinodular goiter |
| TB430 | Thyroid | F | 30y | Graves Disease |
| TB452 | Thyroid | F | 23y | Graves Disease |
| TMB101 | Thymus | M | 7m | Corrective heart surgery |
| TMB117 | Thymus | M | 11y | Corrective heart surgery |
| TMB119 | Thymus | M | 8m | Corrective heart surgery |
| TMB126 | Thymus | F | 1y5m | Corrective heart surgery |
| TMB145 | Thymus | M | 3m | Corrective heart surgery |
| TMB159 | Thymus | F | 8m | Corrective heart surgery |
| TMB016 | Thymus | M | 6y | Corrective heart surgery |
| TMB171 | Thymus | M | 6d | Corrective heart surgery |
| TMB188 | Thymus | M | 6d | Corrective heart surgery |
| TMB195 | Thymus | M | 11m | Corrective heart surgery |
| TMB197 | Thymus | F | 7m | Corrective heart surgery |
| TMB225 | Thymus | F | 3y5m | Corrective heart surgery |
| TMB229 | Thymus | F | 7m | Corrective heart surgery |
| TMB263 | Thymus | F | 4y5m | Corrective heart surgery |
| TMB028 | Thymus | M | 1m | Corrective heart surgery |
| TMB038 | Thymus | M | 7d | Corrective heart surgery |
| TMB004 | Thymus | M | 2m | Corrective heart surgery |
| TMB072 | Thymus | F | 3m | Corrective heart surgery |

Supplementary table 4***.*** *List of samples included in* *allele-specific quantification using NGS (thymus n = 19, thyroid n = 8). The thymus samples were from patients between 0 and 11 years old, obtained mainly after surgery for congenital heart malformation. Thyroid samples were from women between the ages of 20 and 65 years diagnosed with Graves' disease or with multimodular goiter All samples were heterozygous patients for the SNP rs179247.*

**Supplementary table 5**. Primers and probes for ST4 and ST5 relative quantification by Taqman-based quantitative PCR (qPCR).

| **ST4** | Primer Forward | 5’- TTGGAGGAGTATACAGTGGACCAA-3’ |
| --- | --- | --- |
|  | Primer Reverse | 5’- TGGGGCCTTCTGAGTCTCAAA-3’ |
|  | TaqMan Probe | 5’- TTGCTGCTGCCTCTTGGAAGAAAGTCC-3’ |
| **ST5** | Primer Forward | 5’- AAAGATGCATTTGGAGGAGTATACAGT-3’ |
|  | Primer Reverse | 5’- GAACACAGATCCAGGTGCAGTAAGT-3’ |
|  | TaqMan Probe | 5’- CAAGCTTGCTATTCCTAATGAGTCCAT-3’ |
