# Supplementary figures and images for: Regulation of TSHR Expression in the Thyroid and Thymus May Contribute to TSHR Tolerance Failure in Graves' Disease Patients via Two Distinct Mechanisms
Source: Front Immunol. 2019 Jul 18;10:1695. doi: 10.3389/fimmu.2019.01695 (PMC6657650; doi:10.3389/fimmu.2019.01695)

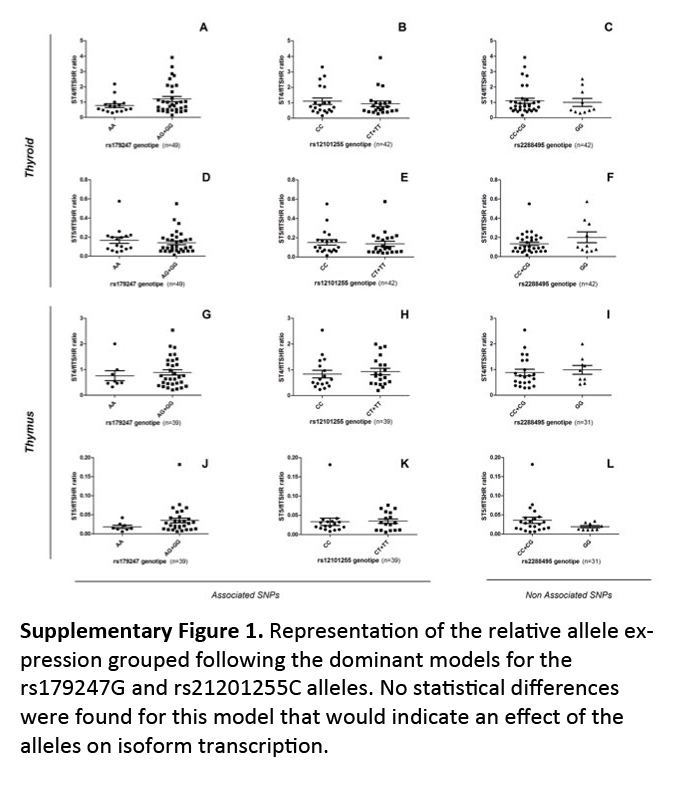

Supplement: Supplementary file 2 [file Image_1.jpeg]

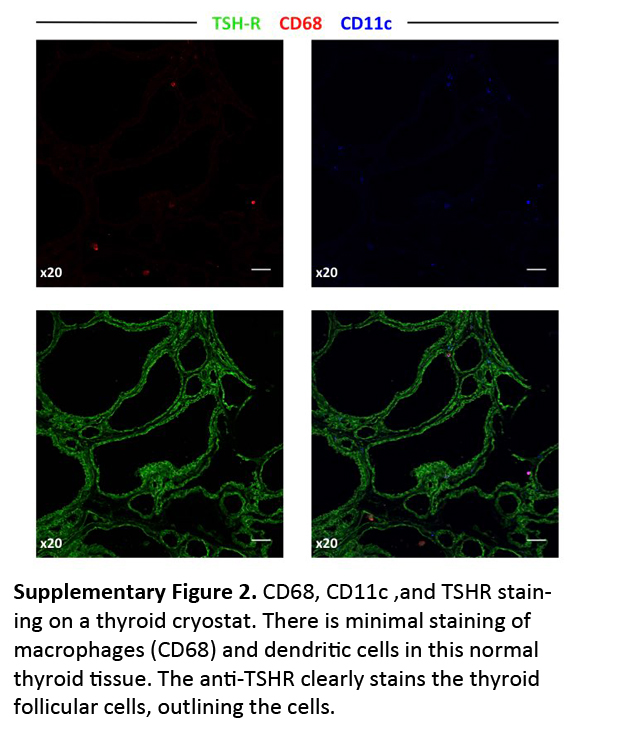

Supplement: Supplementary file 3 [file Image_2.jpeg]
